# Supplementary material for: High-throughput multiplexed serology via the mass-spectrometric analysis of isotopically barcoded beads
Source: Nat Biomed Eng. 2025 Feb 12;9(7):1117–28. doi: 10.1038/s41551-025-01349-0 (PMC12270904; doi:10.1038/s41551-025-01349-0)
Supplement: Supplementary file 1 — Supplementary figures, tables and notes. [file 41551_2025_1349_MOESM1_ESM.pdf]

nature biomedical engineering

Supplementary information

https://doi.org/10.1038/s41551-025-01349-0

High-throughput multiplexed serology via the mass-spectrometric analysis of isotopically barcoded beads

In the format provided by the authors and unedited

Contents

Supplementary Figs. 1–25

Supplementary Notes 1–3

Captions for Supplementary tables 1–4

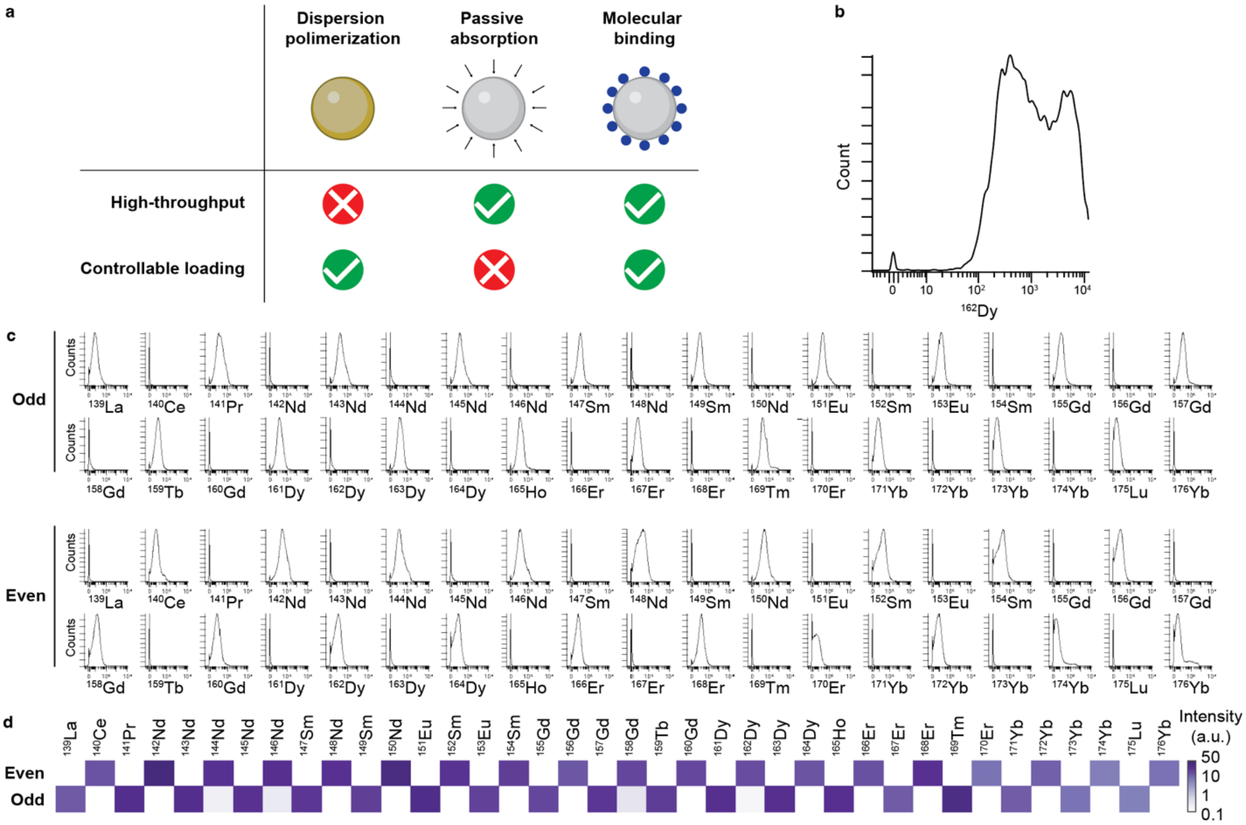

Supplementary Fig. 1 | Comparison of strategies for isotope incorporation on polystyrene beads.

(a) Schematic comparison of strategies for isotope incorporation on polystyrene beads. High-throughput indicates ability to generate tens of thousands of barcode combinations. Controllable loading refers to the ability to load beads with different amounts of isotope and to distinguish amounts based on signal intensity.

(b) Histogram of ^{162}Dy intensity per bead in mono-labeled beads. The isotope was loaded using passive absorption and ^{162}Dy per bead was quantified by mass cytometry.

(c) Histograms of isotope intensity per bead for “odd” and “even” beads. This experiment was performed independently than the one shown in Fig. 1d.

(d) Heatmap of isotope intensities for “odd” and “even” beads shown in (c). Color shows the median of the normalized isotope intensities for odd and even bead populations.

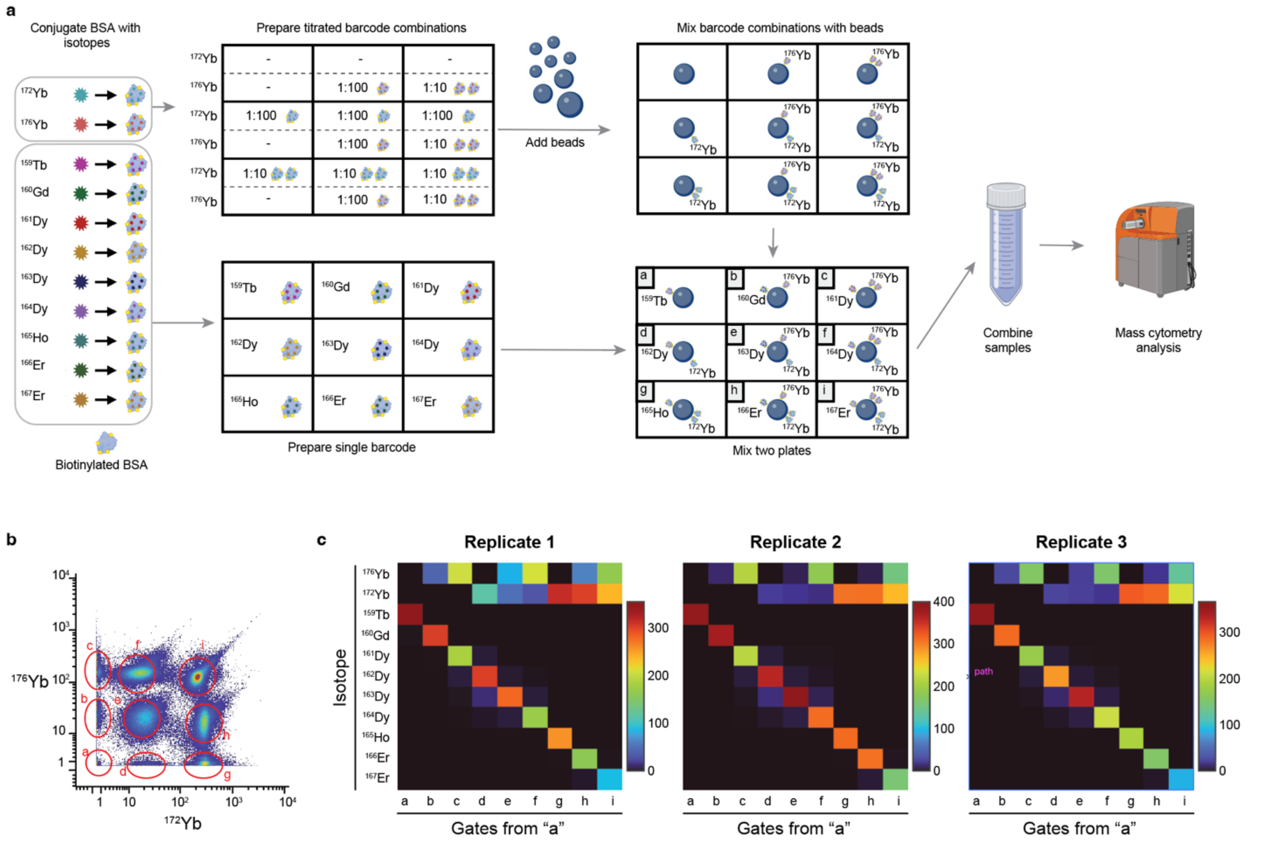

Supplementary Fig. 2 l Isotope loading can be controlled.

(a) Schematic representation of the 9 populations analyzed in the experiment.

(b) Representative dot plot of ^{172}Yb and ^{176}Yb intensities per bead. The red ovals indicate nine distinct populations, each with a different isotope intensity.

(c) Heatmap of isotope intensity in beads loaded with distinct ^{172}Yb and ^{176}Yb intensities. Each of the nine bead populations shown in panel (a) were labeled with another isotope (^{159}Tb to ^{167}Er) to obtain a double barcode that identifies the well of origin for each population. Each row is the intensity of the indicated isotope. Each column is a gate from the population shown in panel (a). Results of three independent experiments are plotted.

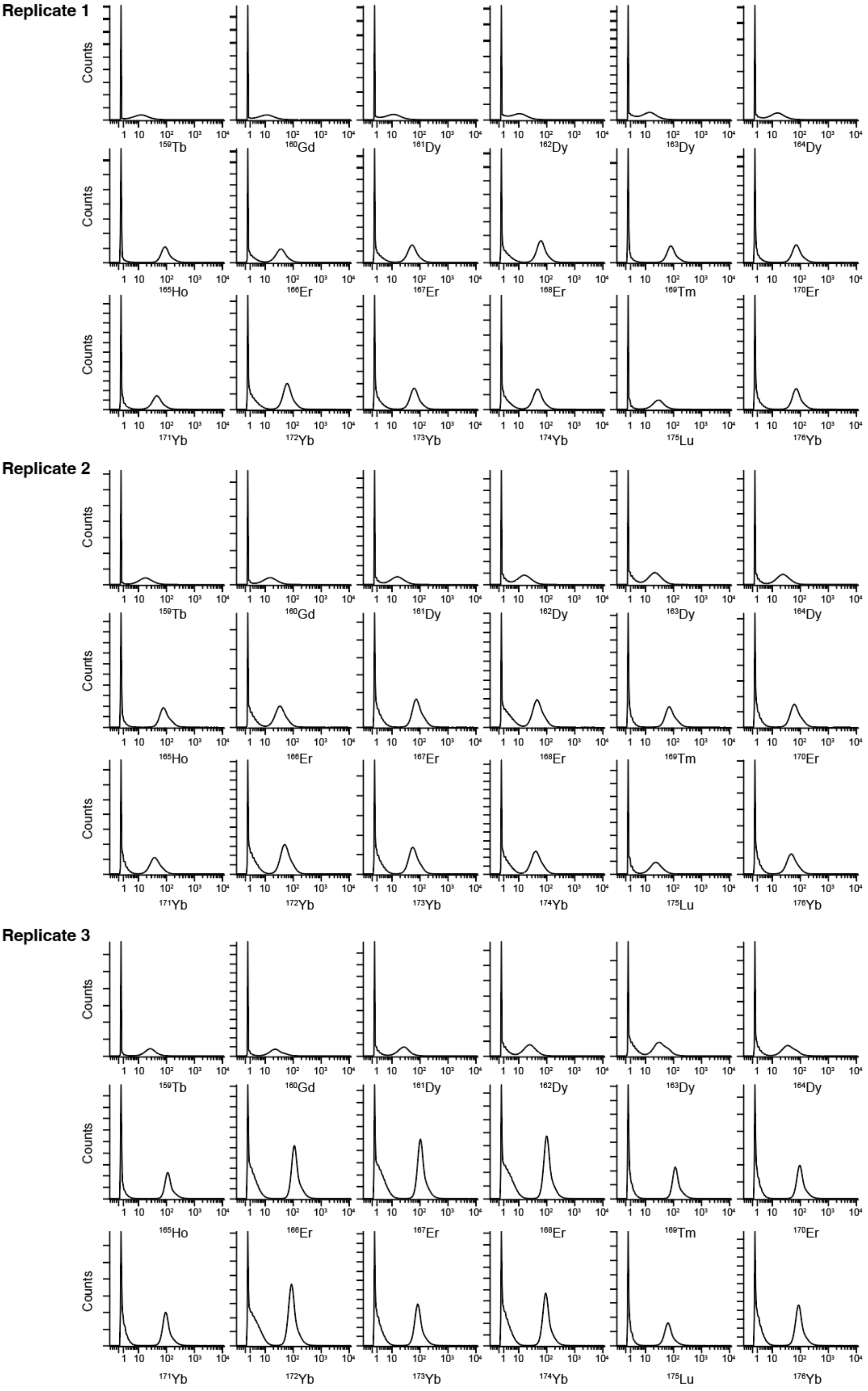

Supplementary Fig. 3 | Histograms of isotope intensity per bead in the bead population consisting of 18,840 barcodes.

Intensities of isotopes ranging from ^{159}Tb to ^{176}Yb. Results of three independent experiments are plotted.

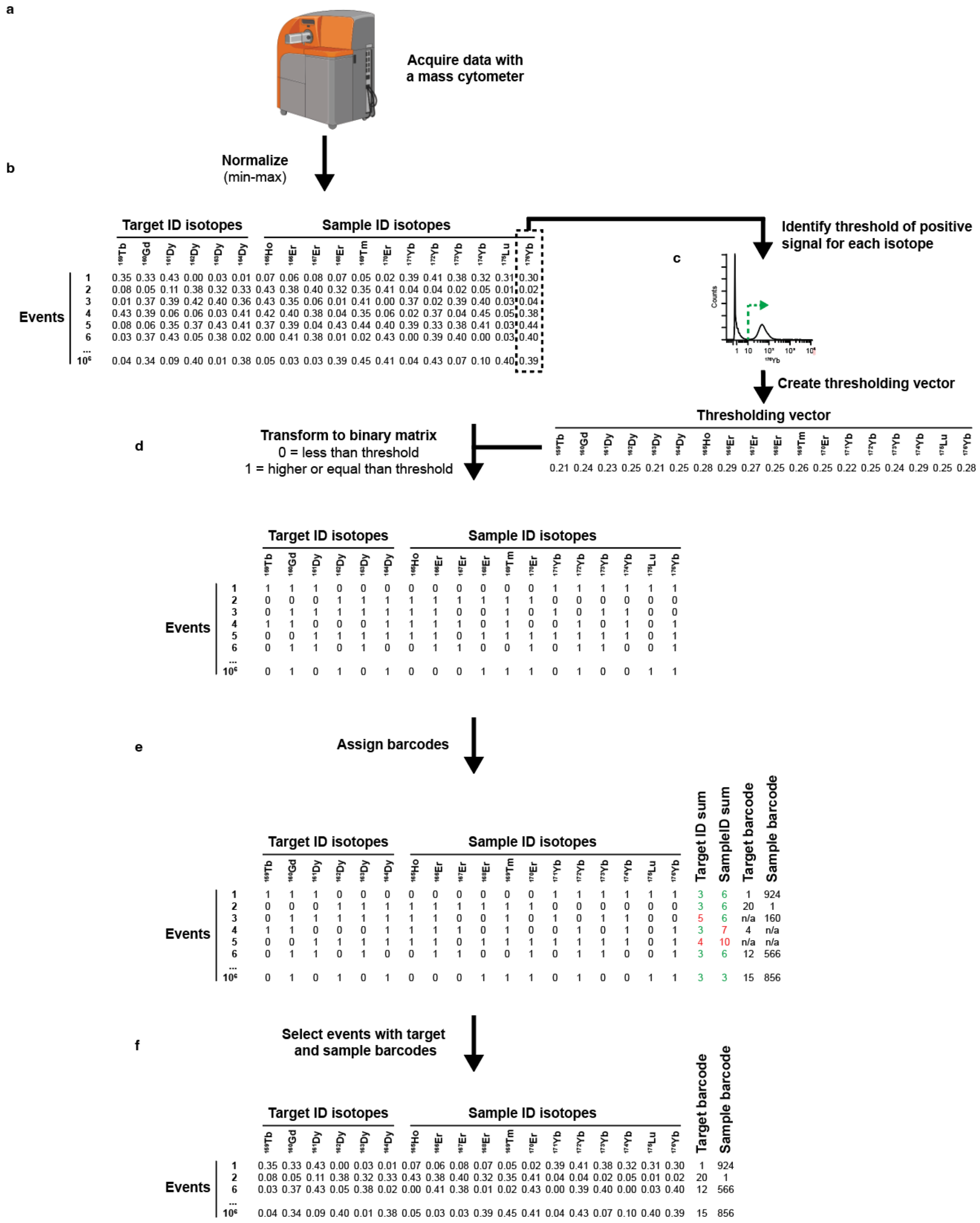

Supplementary Fig. 4 l Schematic of steps in the bead debarcoding pipeline.

(a) Raw data are acquired using a mass cytometer.
(b) Data are arcsine transformed and min-max normalized.
(c) A threshold indicating positive signal is computed for each isotope.
(d) Data are transformed such that each bead has a positive ("1") or negative ("0") isotope signal.
(e) Sample and target IDs are assigned.
(f) Only beads with both sample and target IDs are selected for further analysis. Depicted values are for illustrative purposes and not derived from an experiment.

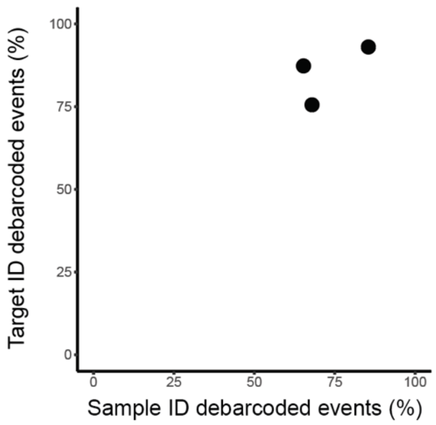

Supplementary Fig. 5 l The debarcoding pipeline identifies beads with correct barcode signatures.

Percentage of events with a correct signature of the target ID barcode set (20 combinations) versus percentage of events with a correct signature of the sample ID barcode set (924 combinations). Data are shown for three independent experiments.

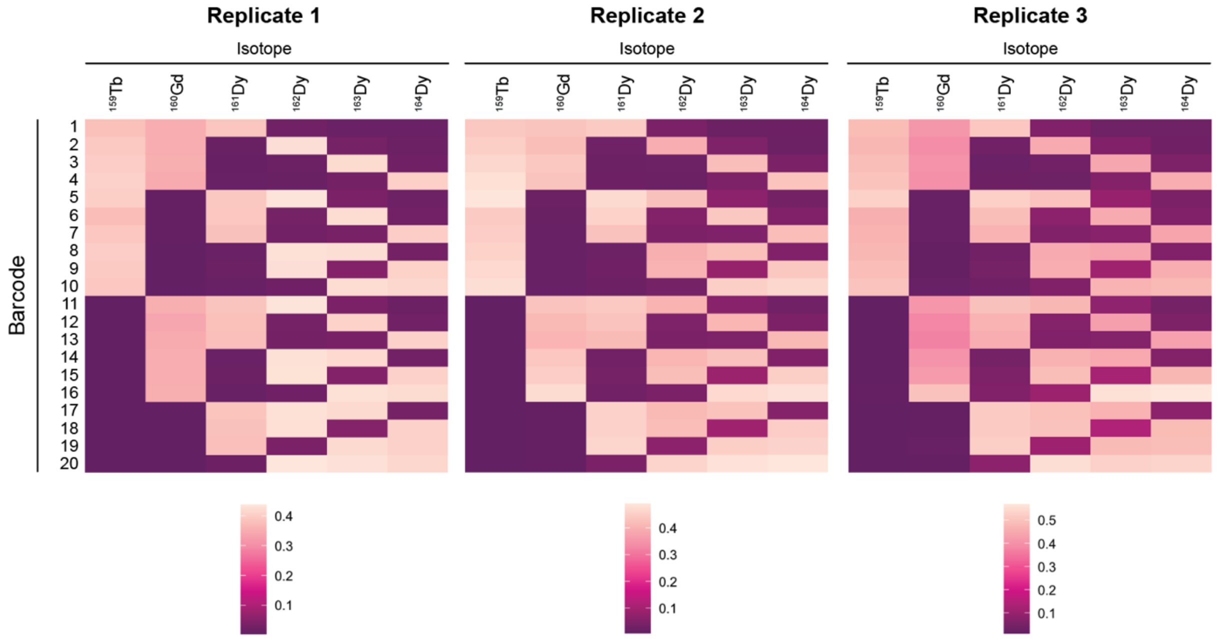

Supplementary Fig. 6 l Isotope signal on each bead population debarcoded by target ID. Measured intensities for isotopes ranging from ^{159}Tb to ^{164}Dy on the 20 indicated bead populations. Color shows the mean of the normalized counts. Data are shown for the three independent experiments.

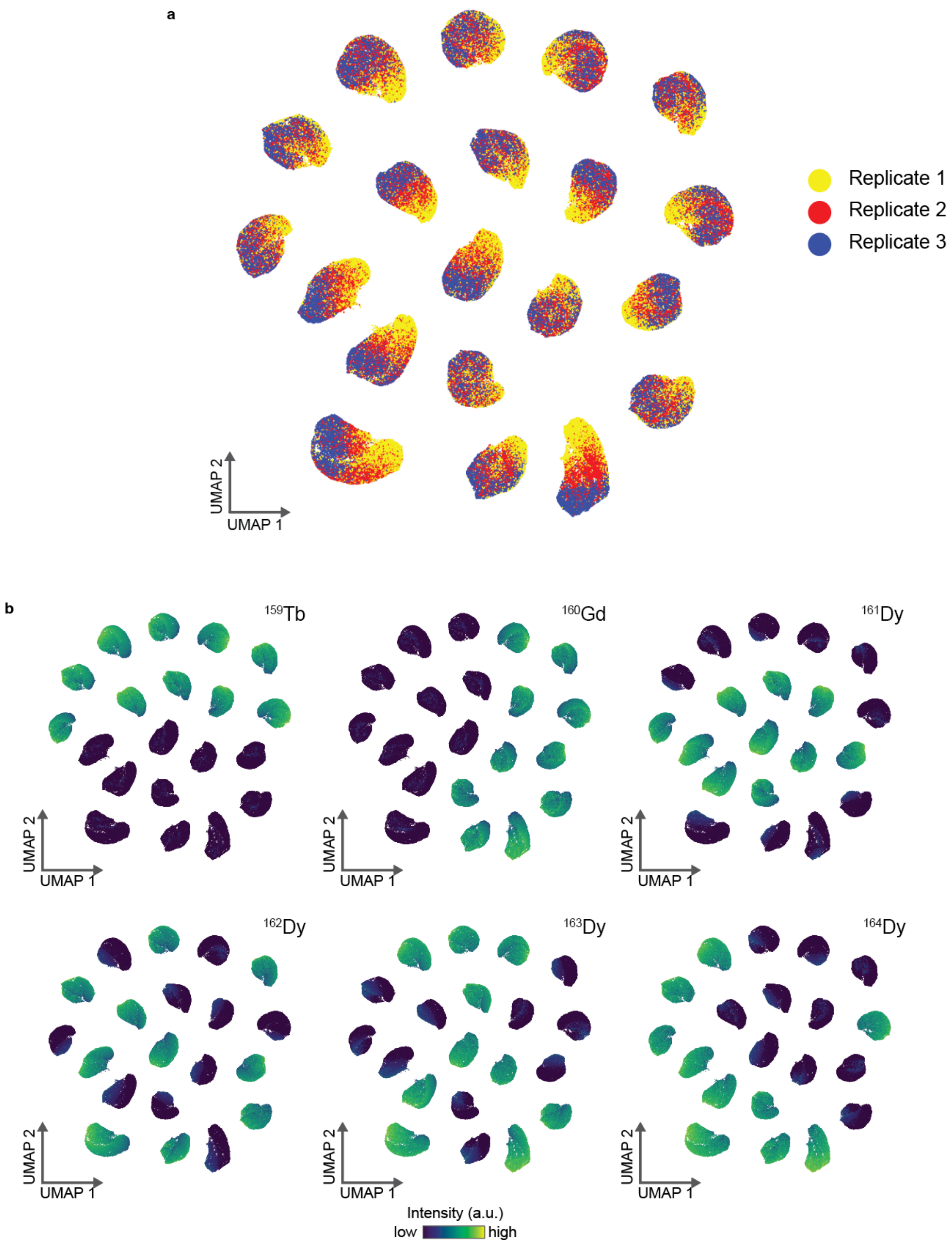

Supplementary Fig. 7 I Generation of 20 distinct isotope-barcoded beads using six isotopes.

(a) UMAP of beads grouped by the intensities of the target ID isotopes, as shown in Fig. 2b, and colored by experimental replicate. Each dot represents a bead.

(b) UMAP of beads grouped by the intensities of the target ID isotopes, as shown in Fig. 2b, and colored by the normalized intensity of the indicated isotope. Each dot represents a bead.

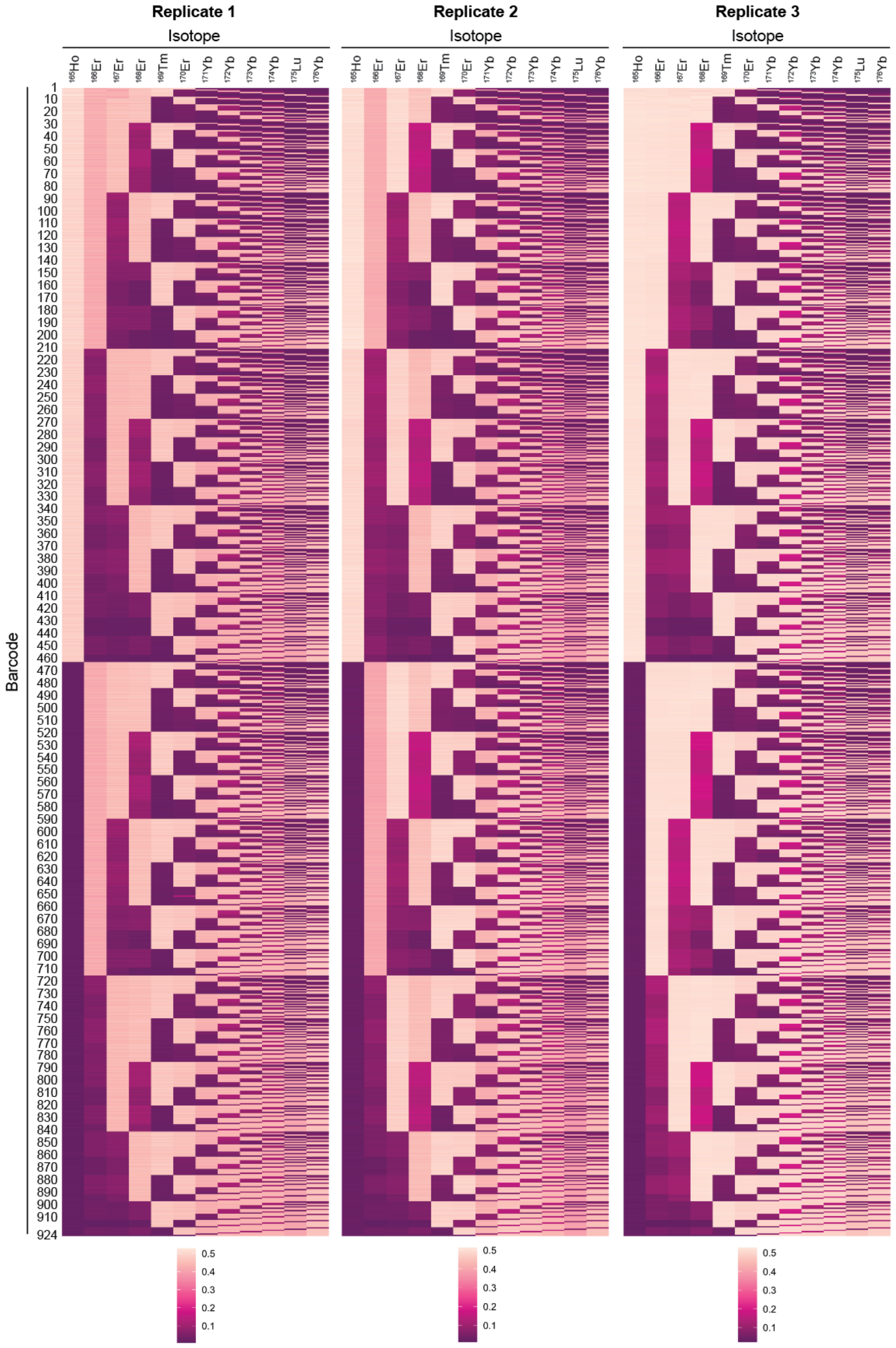

Supplementary Fig. 8 I Isotope signal on each bead population debarcoded by sample ID.

Measured intensities for isotopes ranging from ^{165}Ho to ^{176}Yb on the 924 indicated bead populations. Color shows the mean of the normalized counts. Data are shown for the three independent experiments.

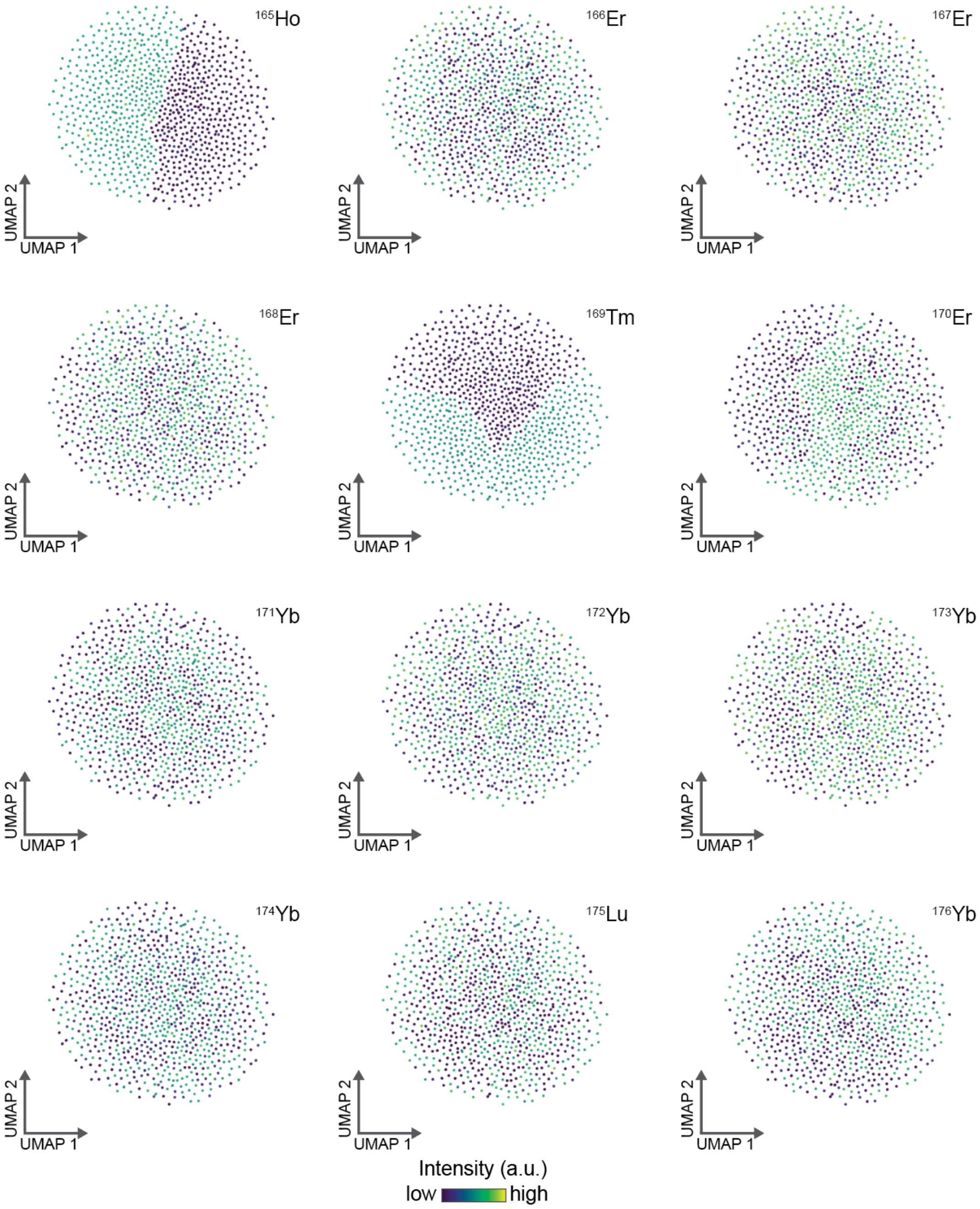

Supplementary Fig. 9 | Generation of 924 distinct isotope-barcoded beads using 12 isotopes.

UMAP of beads from barcode 20 in Fig. 2c grouped by the intensities of the sample ID isotopes and colored by the normalized intensity of the indicated isotope. Each dot represents a bead.

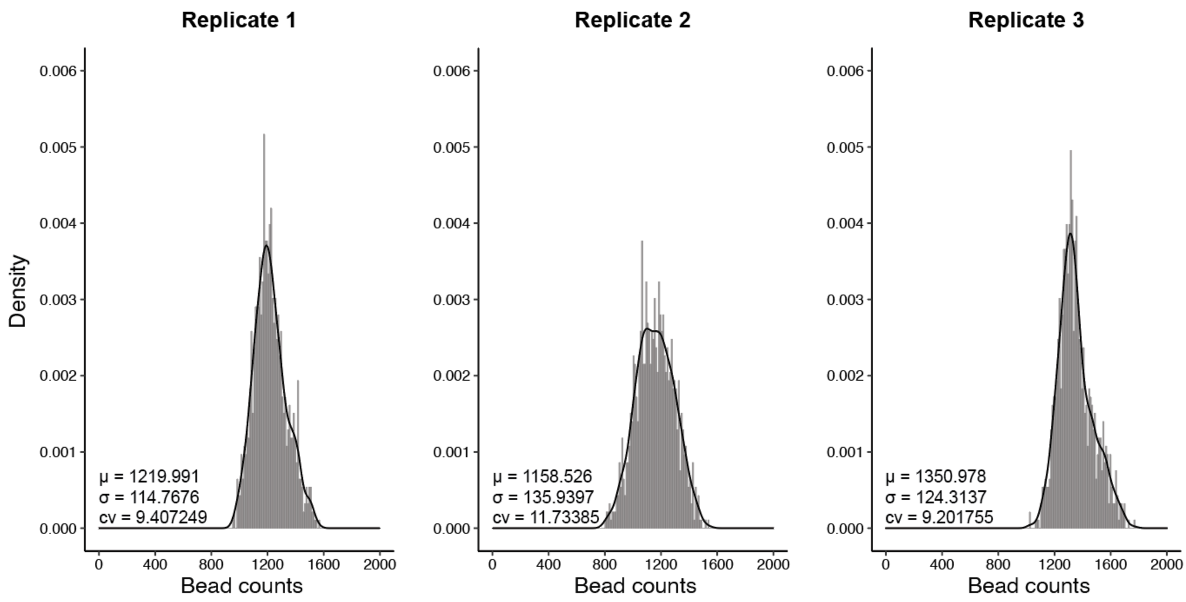

Supplementary Fig. 10 | Bead count per each of the 924 barcodes.

Histograms of bead counts for each sample ID barcode in three independent experiments. The mean, standard deviation, and coefficient of variation is plotted.

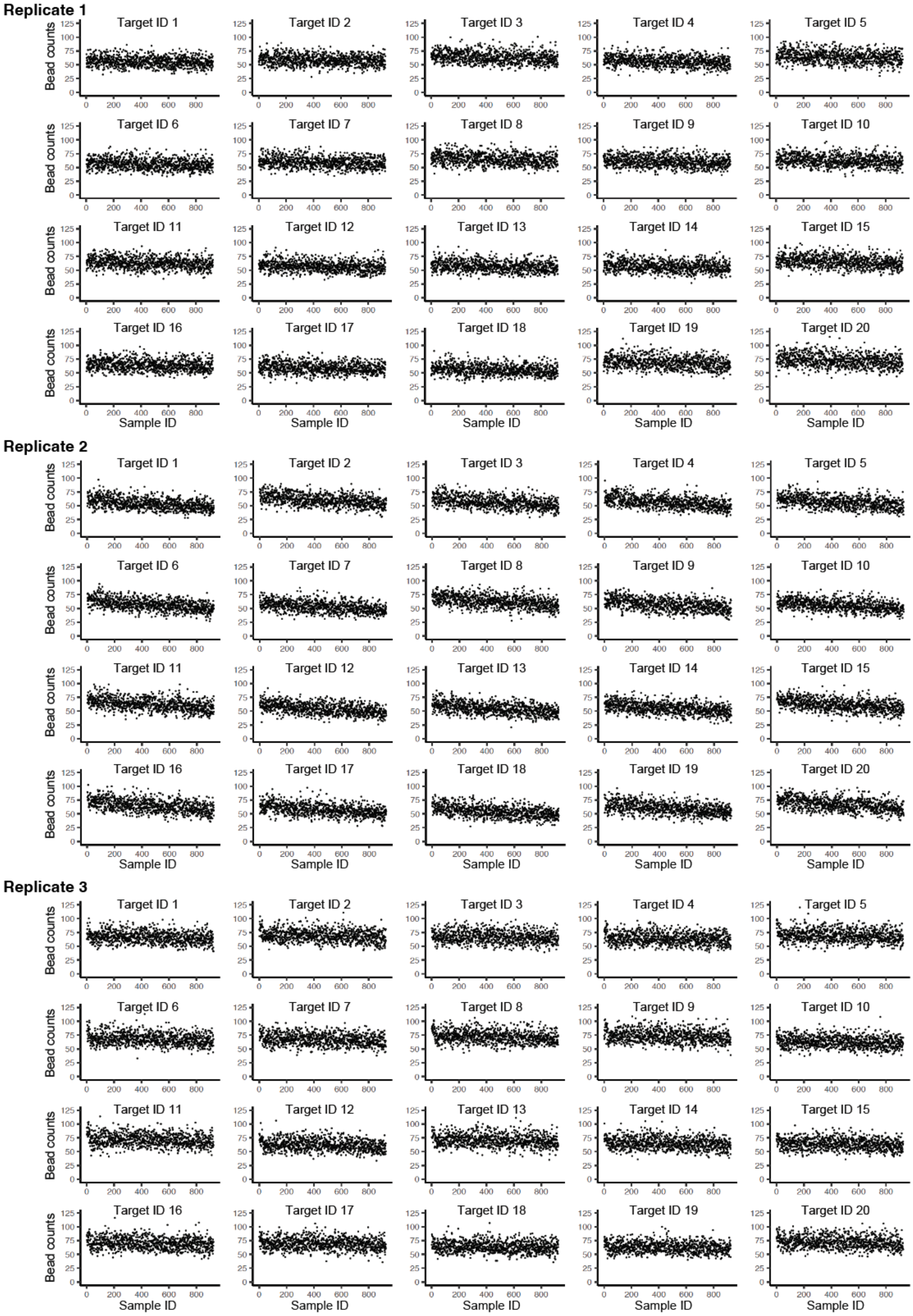

Supplementary Fig. 11 | Bead recovery of 18,480 barcodes.

Bead counts per barcode across 924 Samples IDs in 20 Targets IDs. Data are shown for the three independent experiments.

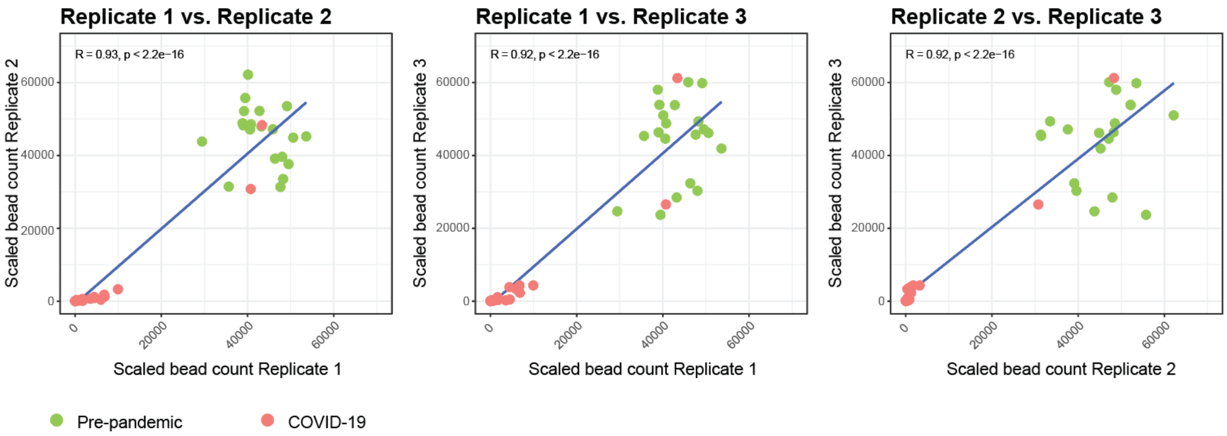

Supplementary Fig. 12 | Reproducibility of the two-bead assay.

Dot plots of the numbers of beads in the flow-through for 20 pre-pandemic and 20 COVID-19 in three independent experiments. Pre-pandemic samples (negative controls) are shown in green and COVID-19-positive samples are shown in red. Samples were randomly distributed in each experiment. Data were scaled to one million total counts per experiment.

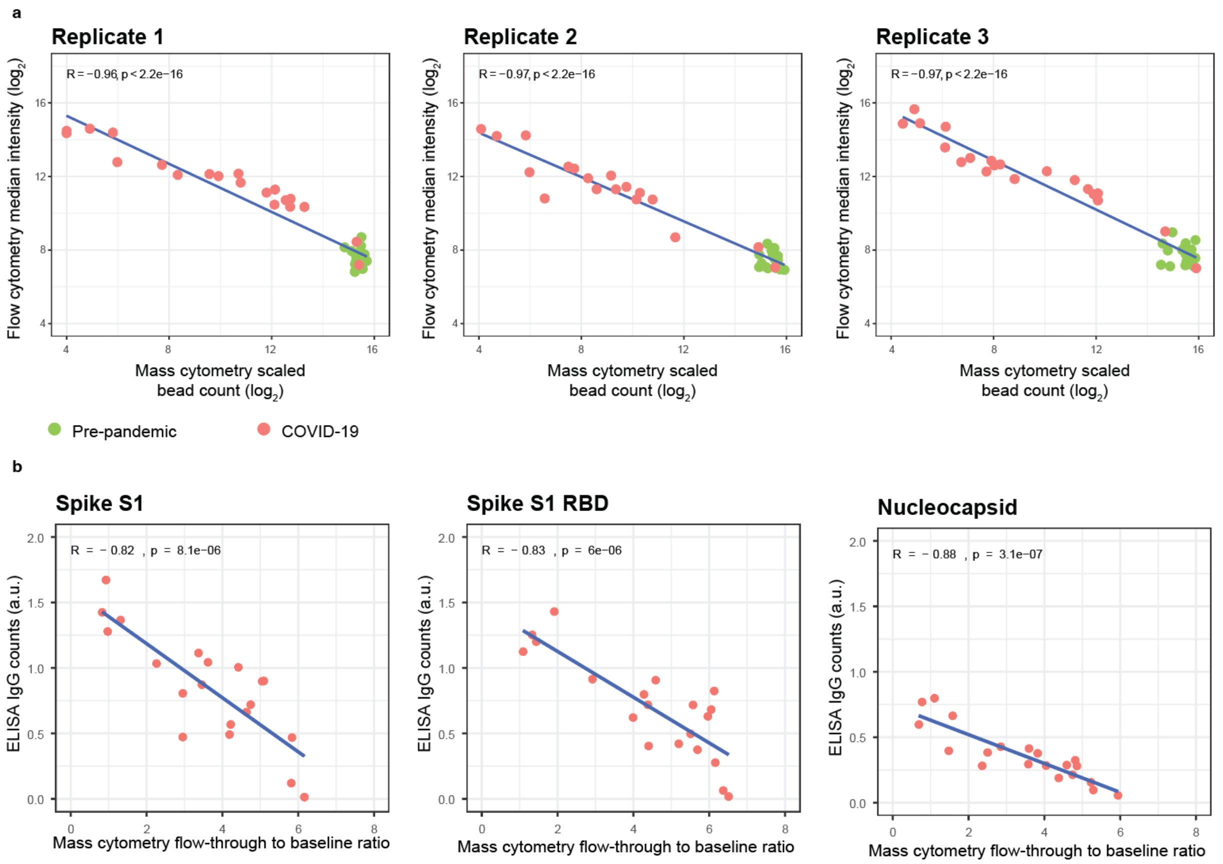

Supplementary Fig. 13 | Individual and pooled analysis of pre-pandemic and COVID-19 samples.

(a) Dot plot of the number of beads in the flow-through of the two-bead mass cytometry assay versus the median of peak intensity per sample of a flow cytometry analysis in three independent experiments. Samples were analyzed individually for the flow cytometry analysis and were pooled for the mass cytometry analysis. Pre-pandemic samples (negative control) are shown in green and COVID-19-positive samples are shown in red. Samples were randomly distributed in each replicate. Flow-through counts were scaled to one million total counts per replicate.

(b) Dot plot of the ratio of beads in the flow-through to baseline of the two-bead mass cytometry assay (X-axis) versus the ELISA values per sample (Y-axis) for Spike S1, Spike S1 RBD, and Nucleocapsid. Samples were analyzed individually for ELISA and were pooled for mass cytometry analysis. Samples were randomly distributed.

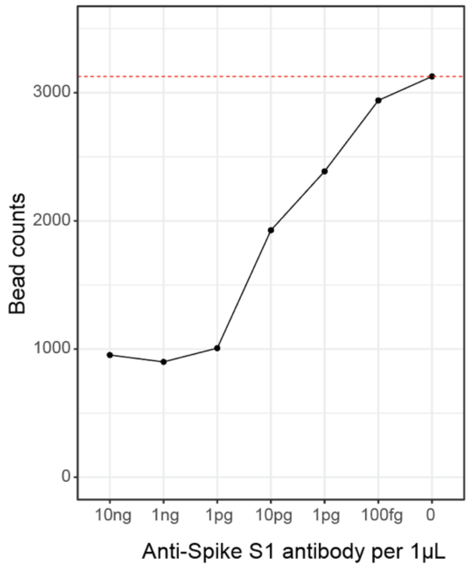

Supplementary Fig. 14 | Limit of detection of the two-bead assay for a SARS-CoV-2 Spike S1 and anti-SARS-CoV-2 Spike S1 IgG pair.

Dot plot of the number of beads in the flow-through when SARS-CoV-2 Spike S1-loaded beads were incubated with antibody concentrations ranging from 0 ng/µL to 10 ng/µL. The analysis was performed on a flow cytometer, analyzing one sample at a time.

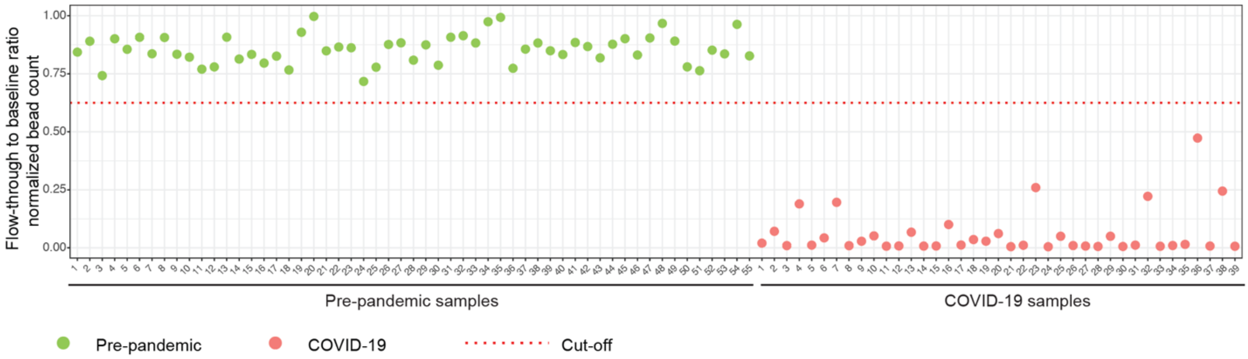

Supplementary Fig. 15 | Sensitivity and specificity assessment of the two-bead assay.

Flow-through to baseline ratio of bead count normalized to non-loaded negative control beads for each sample. Pre-pandemic samples (negative control) are shown in green and COVID-19-positive samples are shown in red.

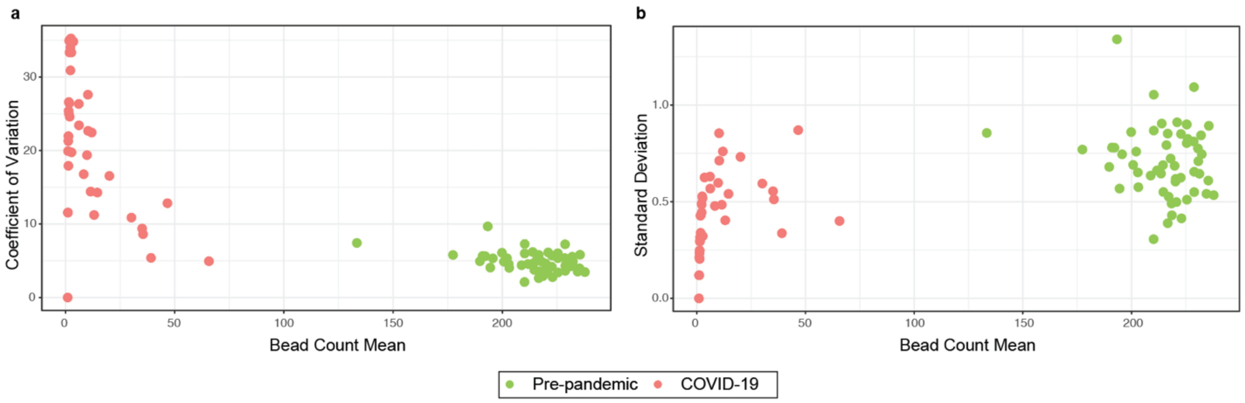

Supplementary Fig. 16 | Reproducibility of the two bead-assay assessed by the coefficient of variation and standard deviation of replicates.

(a) Coefficient of variation for pre-pandemic and COVID-19-positive samples versus bead count mean. Square root normalization was applied due to low absolute values of the data.

(b) Standard deviation across pre-pandemic and COVID-19-positive samples versus bead count mean. Square root normalization was applied due to low absolute values of the data.

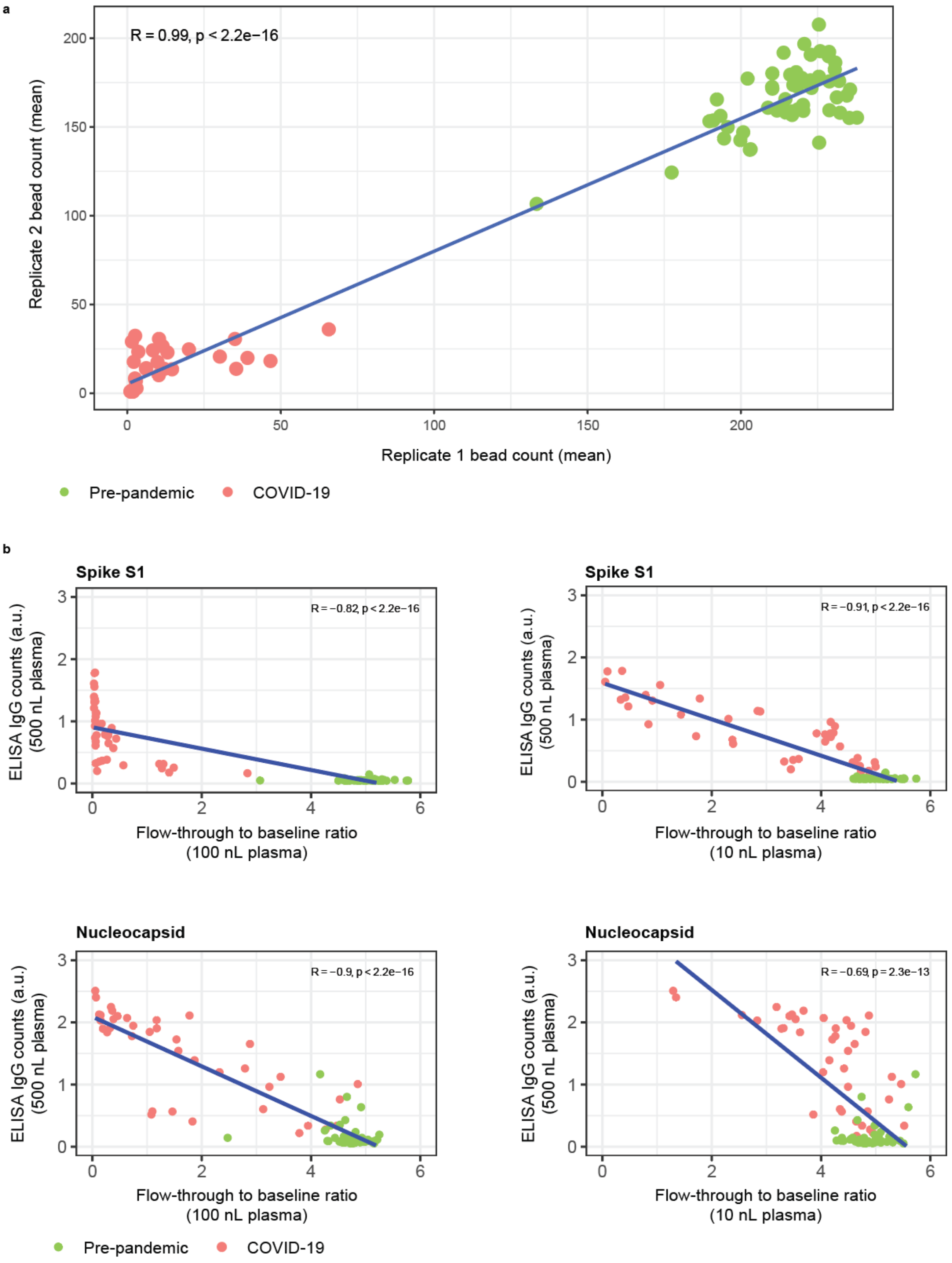

Supplementary Fig. 17 | Reproducibility of the two-bead assay in 55 pre-pandemic and 39 COVID-19 samples and individual and pooled analysis of COVID-19 samples.

(a) Dot plot of the bead count per sample in two independent two-bead assays for a set of 55 pre-pandemic negative samples (green dots) and 39 COVID-19-positive samples (red dots). Pearson correlation is 0.99.

(b) Dot plot of the ratio of beads in the flow-through to baseline of the two-bead mass cytometry assay (X-axis) versus the ELISA values per sample (Y-axis) for Spike S1 and Nucleocapsid. Samples were analyzed individually for ELISA and were pooled for mass cytometry analysis. Samples were randomly distributed.

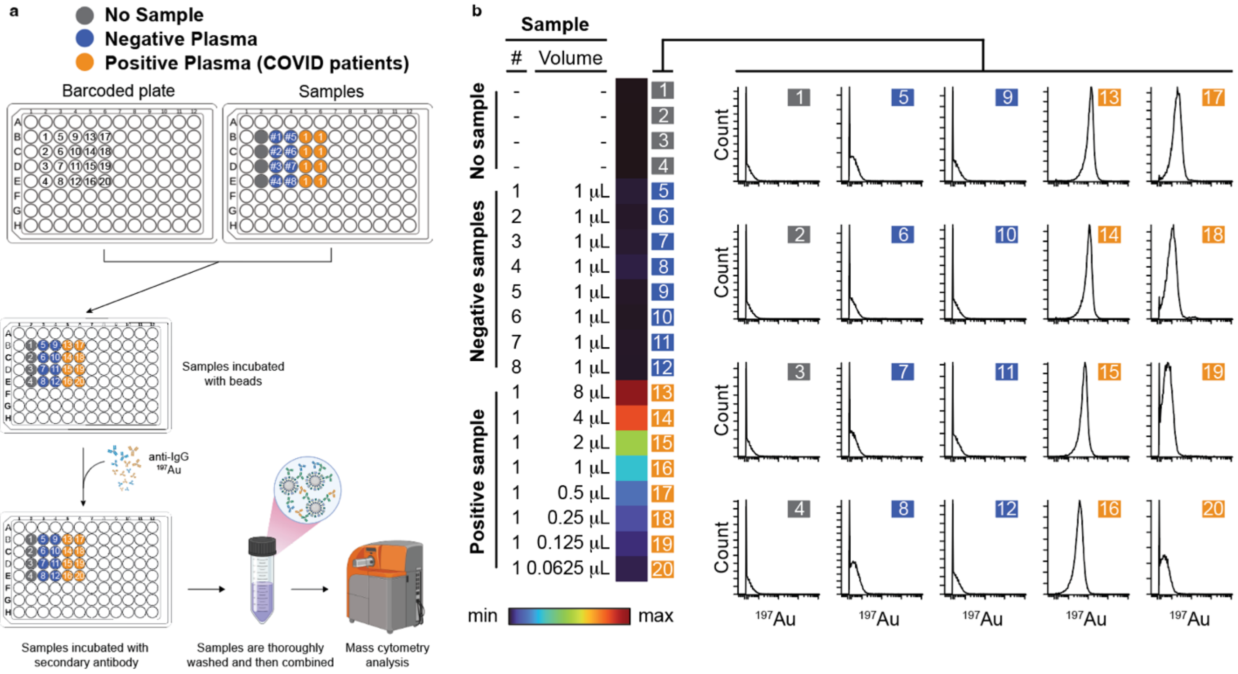

Supplementary Fig. 18 | Validation of secondary antibodies for the quantification of immunoglobulins on isotope-barcoded beads.

(a) Schematic representation of the secondary antibody validation experiment.

(b) Heatmap and histograms of IgG levels on Spike S1-loaded, isotope-barcoded beads incubated with plasma samples from one COVID-19 positive sample (titrated from 8 µL to 0.0625 ☐L) and eight plasma samples collected prior to the COVID-19 pandemic (1 µL each). IgG levels on beads were quantified by mass cytometry using anti-human IgG conjugated to gold nanoparticles.

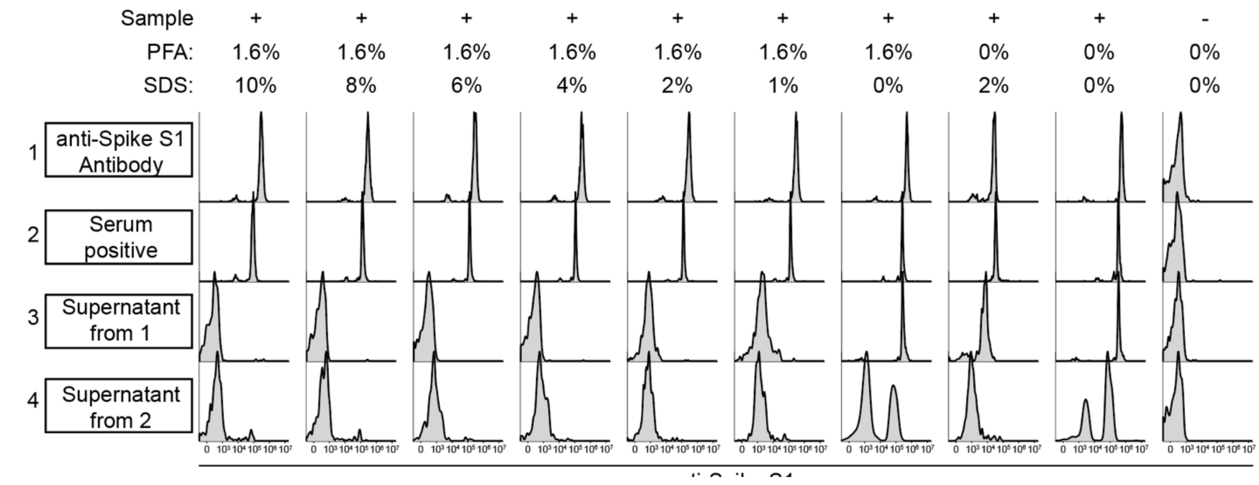

Supplementary Fig. 19 | Validation of a wash free step.

Spike S1-loaded beads were incubated with monoclonal anti-Spike S1 antibody or a COVID-19-positive serum sample. After incubation, samples were treated with PFA and, subsequently, SDS. Supernatants were collected and tested for binding to a new set of beads. Histograms show IgG levels on 1) beads incubated with monoclonal anti-Spike S1 antibody, 2) bead incubated with a COVID-19-positive serum sample, 3) supernatant from the monoclonal anti-Spike S1 antibody sample, and 4) supernatant form the COVID-19-positive serum sample after treatment with indicated percentages of PFA and SDS.

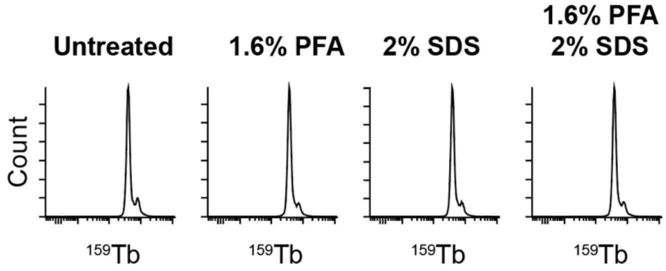

Supplementary Fig. 20 | Validation of isotope levels by the wash-free chemical treatment.

Histogram of ^{159}Tb intensity per bead on mono-labeled beads incubated with 1.6% PFA, 2% SDS, or both PFA and SDS. ^{159}Tb per bead was quantified by mass cytometry.

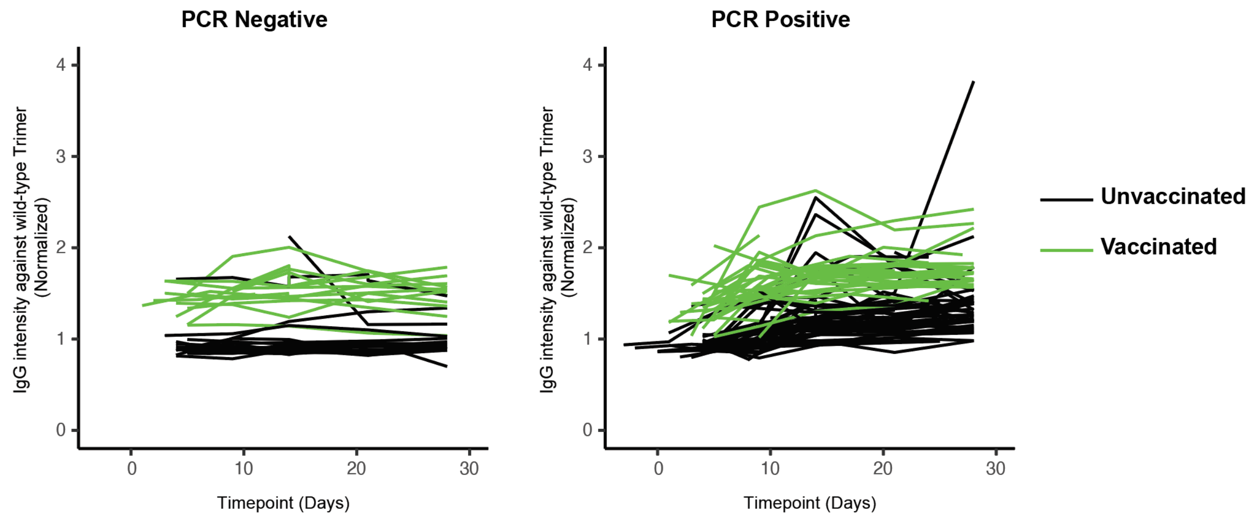

Supplementary Fig. 21 | Overview of seroconversion against the wild-type SARS-CoV-2 Trimer in the analyzed samples from the FIND study.

Line plots of the IgG levels against SARS-CoV-2 Spike Trimer per individual as a function of time after onset of symptoms. Samples were classified by PCR result, and lines are colored by vaccination status. Each line represents an individual.

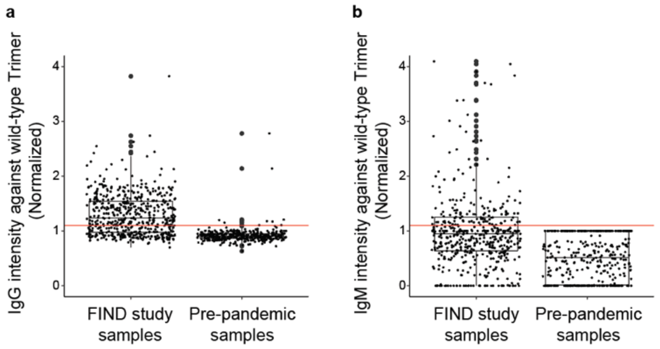

Supplementary Fig. 22 | Sensitivity of mass cytometry assay in 100 pre-pandemic samples.

Boxplot of the (a) IgG and (b) IgM levels against SARS-CoV-2 Spike Trimer in the samples from the FIND study and 100 pre-pandemic samples, each assessed in triplicate or quadruplicate for a total of 359 test. The red line indicates the threshold. Nine samples (2.51%) were above the threshold for IgG and zero samples (0%) were above the threshold for IgM.

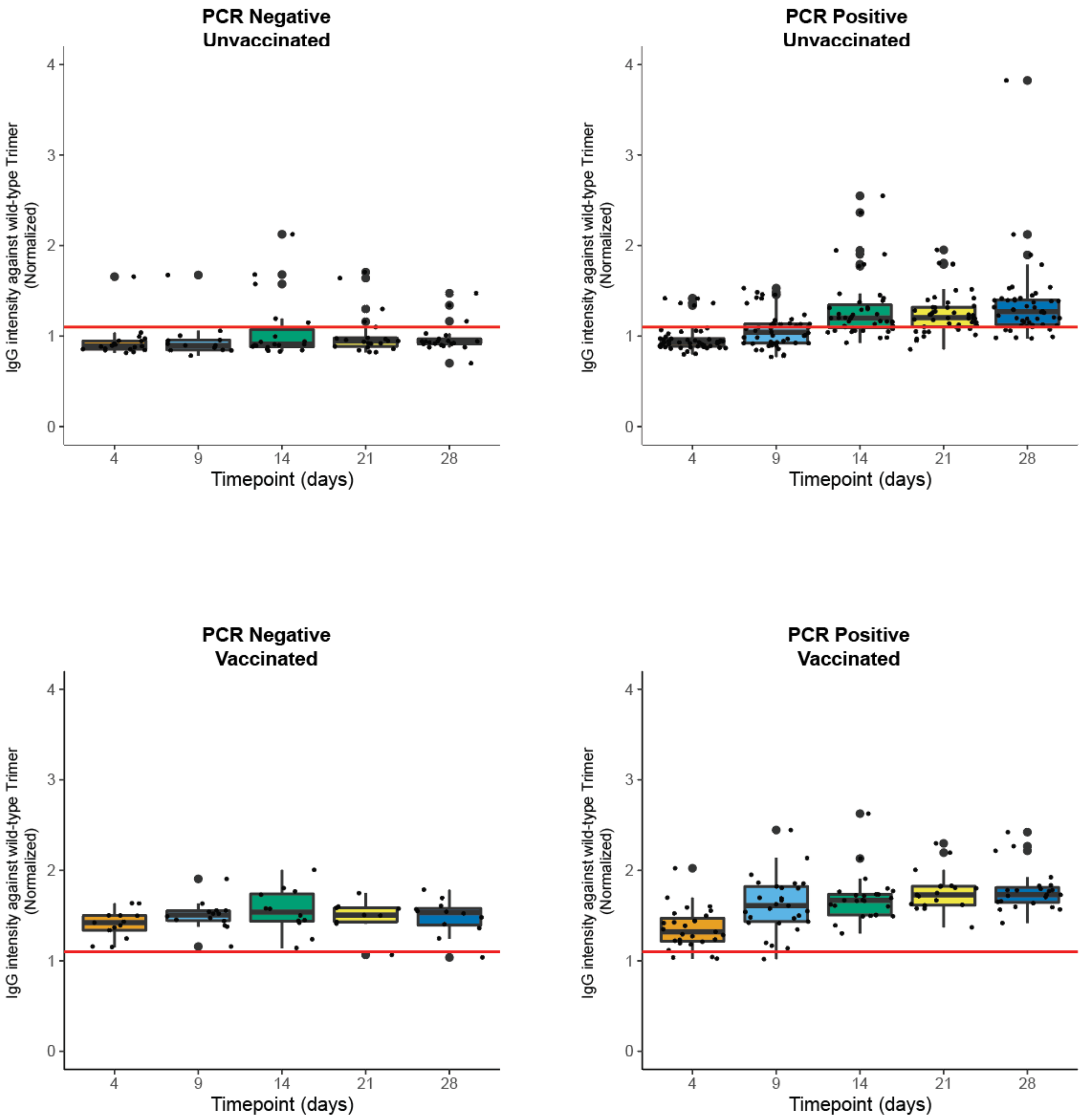

Supplementary Fig. 23 | Comparison of IgG levels at distinct timepoints by vaccination and infection status.

Boxplot of the IgG levels against SARS-CoV-2 Spike Trimer per sample by time after onset of symptoms or vaccination. Samples are classified by vaccination status and PCR results. Each dot represents a sample.

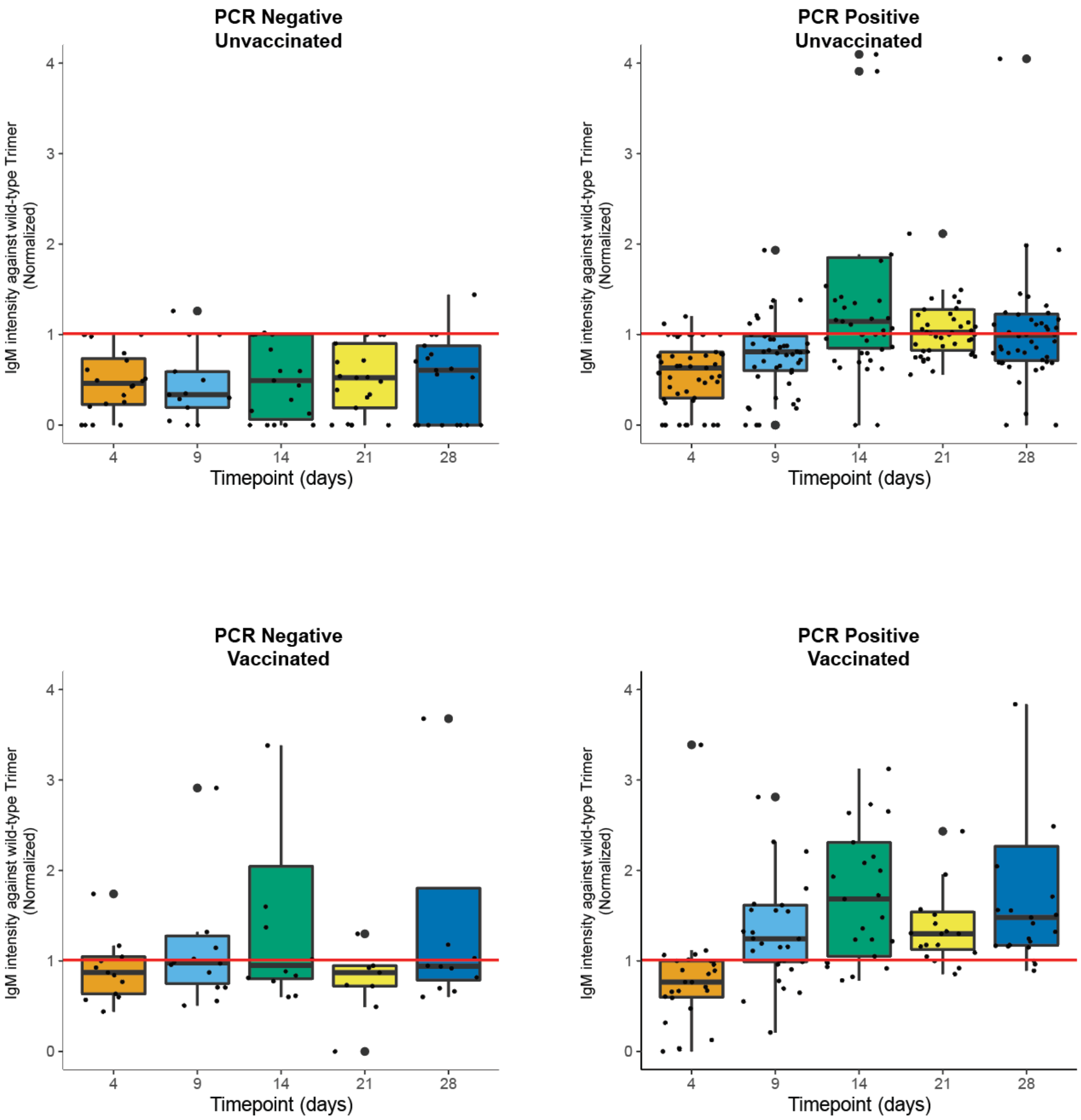

Supplementary Fig. 24 | Comparison of IgM levels at distinct timepoints by vaccination and infection status.

Boxplot of the IgM levels against SARS-CoV-2 Spike Trimer per sample by time after onset of symptoms or vaccination. Samples are classified by vaccination status and PCR results. Each dot represents a sample.

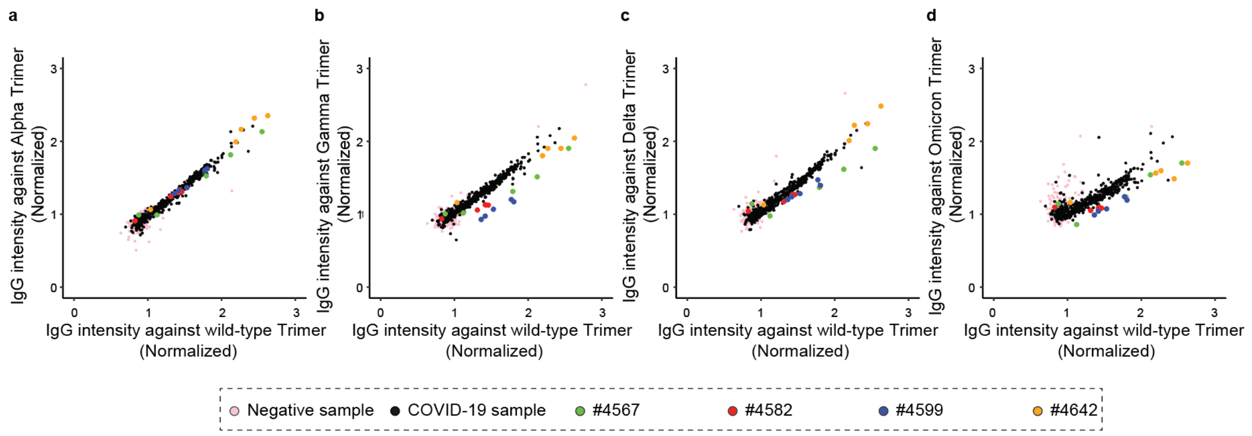

Supplementary Fig. 25. Correlation of IgG levels per sample to distinct variants.

Dot plots of the levels of IgG against (a) Alpha, (b) Gamma, (c) Delta, and (d) Omicron Spike Trimers versus levels of wild-type SARS-CoV-2 Spike Trimer. Each dot represents a sample.

<u>Supplementary Note 1:</u> **Cost of materials for each mass cytometry run of 924 samples and 20 targets**

Multiplex serology by mass spectrometry analysis of beads is a cost-effective technology at large number of samples and targets per run. For instance, if we take a conservative estimate, it would be feasible implementing 100 runs per mass cytometer per year (approximately 2 runs of 4 to 6 hours per week). In this scenario, the cost of materials for each run is estimated to be $1,908.55:

Each run could accommodate 924 samples (including controls), and therefore the cost of materials per

Number of tests per sample	20
Samples per assay (samples + internal assay QC)	924
Assays per instrument each year	100
Isotopes for sample barcode	12
Isotopes for target barcode	6

Item	Cost per unit	mber of 924 assays per ur	ber of samples per	Cost per sample	Units needed per year	Cost per year	Notes
Functionalized Polystyrene beads	$245.00	5.00	4620	$0.0530	20	$4,900.00	https://www.spherotech.com/coa_pol_par.htm
96 well liquid handler tips (10 pack)	$60.00	1.00	924	$0.0649	100	$6,000.00	https://www.amazon.com/200ul-Pipette-Tips-Universal-Racks%EF%BC%88960/dp/B098D489F2/ref=pd_lpo_sccl_3/142-0267336-
Bravo tips (pack of 50 boxes)	$450.00	5.00	4620	$0.0974	20	$9,000.00	https://www.agilent.com/en/product/automated-liquid-handling/consumables-for-lab-automation/bravo-lab-disposable-pipette-tips
96 well plates (50 pack)	$400.00	2.50	2310	$0.1732	40	$16,000.00	https://ecatalog.corning.com/life-sciences/b2b/CA/en/Microplates/Assay-Microplates/96-Well-Microplates/Corning%C2%AE-96-well-
Anti-IgG-Gold	$401.00	10.00	9240	$0.0434	10	$4,010.00	https://www.nanoprobes.com/products/Nanogold-Antibody-Conjugates.html
Lancets	$13.00	0.11	100	$0.1300	924	$12,012.00	https://www.amazon.com/McKesson-16-PBSL28G-Safety-Lancet-Needle/dp/B01N704MMD/ref=sr_1_5?crid=213GCFLKC1WGQ&
Whatmann blood collection	$45.20	0.54	500	$0.0904	184.8	$8,352.96	https://www.sigmaaldrich.com/US/en/product/aldrich/wha10010155
Chemicals (PBS, Tween 20, BSA, water)	$1,000.00	50.00	46200	$0.0216	2	$2,000.00	Variety of cheap chemicals
BSA-biotin	$129.00	5.00	4620	$0.0279	20	$2,580.00	https://www.sigmaaldrich.com/US/en/product/sigma/a8549
Consumables (eppendorfs, falcon tubes, tips)	$1,000.00	50.00	46200	$0.0216	2	$2,000.00	Variety of cheap consumables
Tempest (Chips)	$12,000.00	100.00	92400	$0.1299	1	$12,000.00	https://wcm.formulatrix.com/product/tempest-chip-silicone-hv-1ul-5ul-pack-of-6/
Tempest (Other consumables)	$2,000.00	100.00	92400	$0.0216	1	$2,000.00	https://wcm.formulatrix.com/product-category/tempest/
Isotope conjugation kit (for patient barcode)	$1,200.00	2.00	1848	$0.6494	50	$60,000.00	https://fluidigm.my.site.com/Storefront/Cytometry/ConsumablesandReagentsCytometry/MaxparAntibodyLabelingKits?cclcl=en_US
Isotope conjugation kit (for sample barcode)	$600.00	2.00	1848	$0.3247	50	$30,000.00	https://fluidigm.my.site.com/Storefront/Cytometry/ConsumablesandReagentsCytometry/MaxparAntibodyLabelingKits?cclcl=en_US
Protein (each)	$500.00	50.00	46200	$0.0108	40	$20,000.00	Assuming $500 per 100 ug of protein; prices might vary
						$190,854.96	Total cost
						$1,908.55	Cost per assay
						$2.07	Cost per sample
						$0.10	Cost per test in sample

sample is estimated to be $2.07. Each sample would be tested for up to 20 targets and therefore the cost of materials is estimated to be <u>$0.10 per analyte per sample</u>. These cost estimates do not include operational and instrumental costs, which would be the capital and annual maintenance costs of a mass cytometer, the annual full-time salaries of one or two technicians, amongst others.

Supplementary Note 2: **Step-by-step illustration of bead generation, assay, and analysis**

Step 1: Generation of isotope-conjugated BSA. A set of 18 distinct isotopes are individually conjugated to biotinylated BSA. We typically use two types of barcoding:

1) Target ID barcoding, which contains isotopes from ^{159}Tb to ^{164}Dy to generate 20 unique combinations;
2) Sample ID barcoding, which uses isotopes from ^{165}Ho to ^{176}Yb to generate 924 unique combinations.

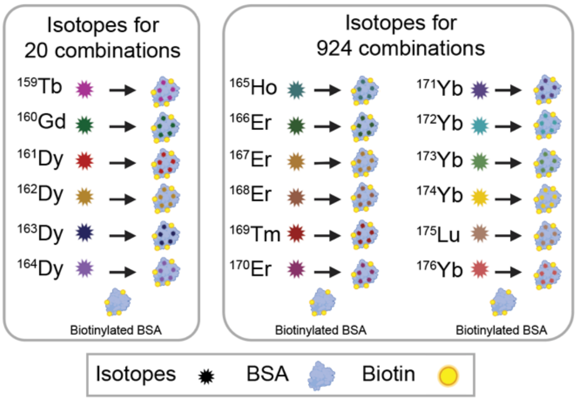

Step 2: Loading of antigens onto beads. Each selected Avi-tagged protein is loaded onto streptavidin beads.

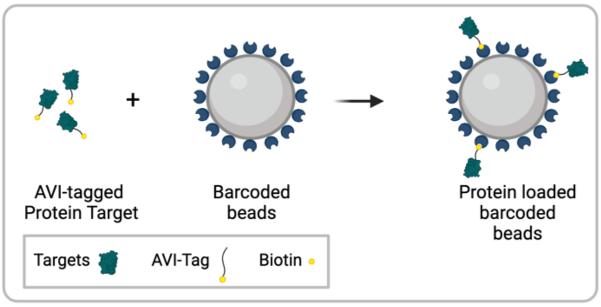

Step 3: Generation of 20 barcodes for Target ID. By selecting 3 out of the 6 isotope-conjugated BSA proteins from *Step 1*, we generate all 20 possible barcode combinations. These combinations are then added to streptavidin-coated beads, containing Avi-tagged proteins of interest. Thus, we ensure that each barcode is paired with a unique protein.

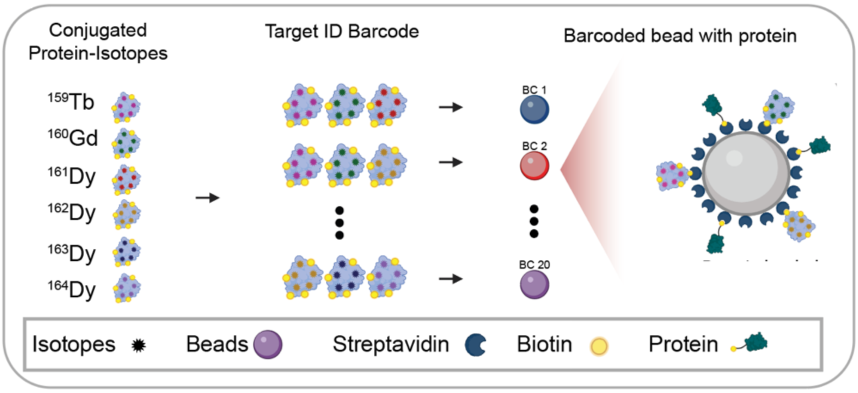

Step 4: Pooling of beads and plate distribution. The 20 barcoded beads loaded with a protein target are pooled and aliquoted to 10 96-well plates.

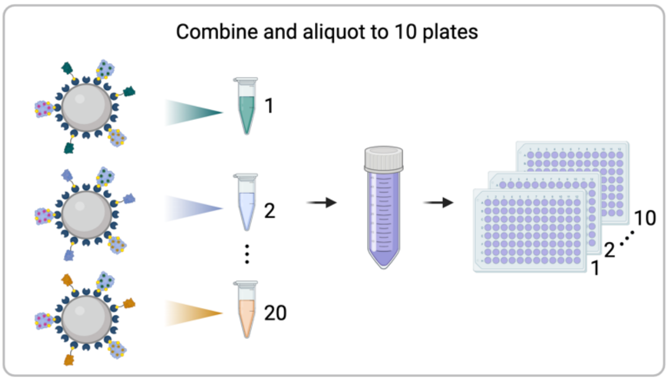

Step 5: Generation of 924 barcodes for Sample ID. By selecting 6 out of the 12 isotope-conjugated BSA proteins from *Step 1***,** we generate all 924 possible barcode combinations. BSA combinations are added to the 10 96-well plates, one combination on each well. This process is automated using liquid handlers and plates are stored at 4° C until use.

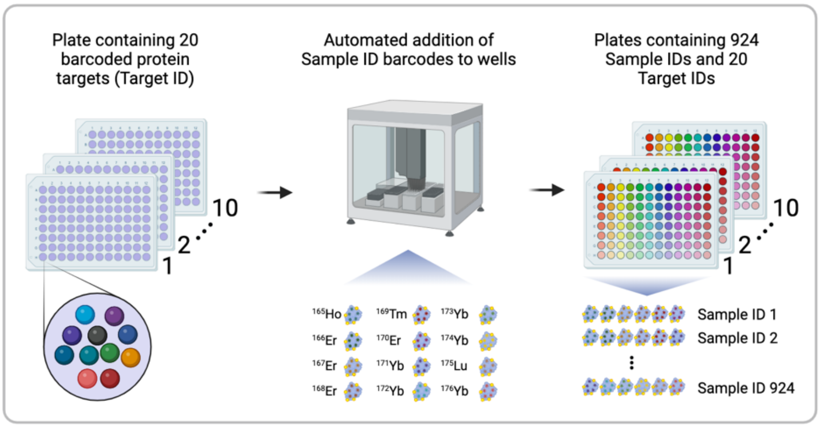

Step 6: Incubation of plasma samples with barcoded beads. Samples are incubated with barcoded beads in 96-well plates.

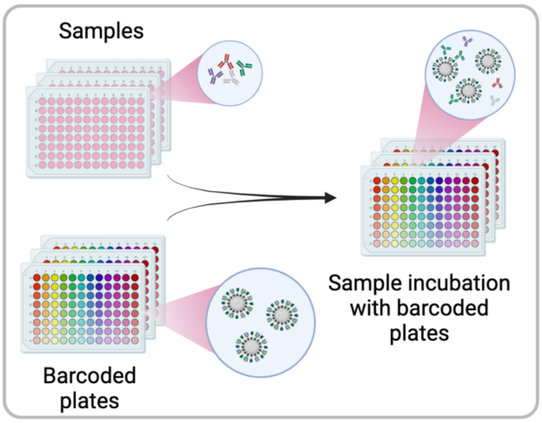

Step 7: Sample fixation and denaturation. The samples are sequentially fixed with PFA and then denatured with SDS.

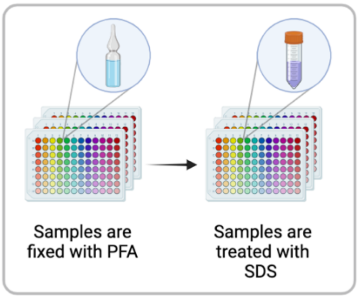

Step 8: Secondary antibody labeling and mass cytometry acquisition. Samples are combined in one tube and thoroughly washed. Secondary antibodies, e.g. anti-IgG conjugated to ^{197}Au, are added to the mixture. After incubation, the pooled sample is washed and analyzed in a mass cytometer.

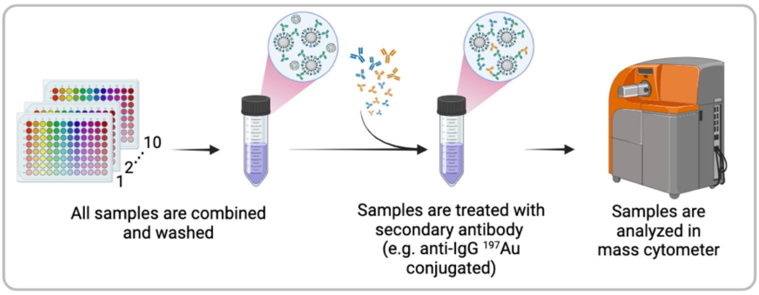

Step 9: Bead debarcoding. The data acquired from the mass cytometer are max-min normalized and a threshold value is identified for each isotope. Based on the threshold, the beads are assigned a Target and a Sample ID.

Data

Events	Target ID isotopes						Sample ID isotopes											
	^{159}Tb	^{160}Gd	^{161}Dy	^{162}Dy	^{163}Dy	^{164}Dy	^{165}Ho	^{166}Er	^{167}Er	^{168}Er	^{169}Tm	^{170}Er	^{171}Yb	^{172}Yb	^{173}Yb	^{174}Yb	^{175}Lu	^{176}Yb
1	0.35	0.33	0.43	0.00	0.03	0.01	0.07	0.06	0.08	0.07	0.05	0.02	0.39	0.41	0.38	0.32	0.31	0.30
2	0.08	0.05	0.11	0.38	0.32	0.33	0.43	0.38	0.40	0.32	0.35	0.41	0.04	0.04	0.02	0.05	0.01	0.02
3	0.01	0.37	0.39	0.42	0.40	0.36	0.43	0.35	0.06	0.01	0.41	0.00	0.37	0.02	0.39	0.40	0.03	0.04
4	0.43	0.39	0.06	0.06	0.03	0.41	0.42	0.40	0.38	0.04	0.35	0.06	0.02	0.37	0.04	0.45	0.05	0.38
5	0.08	0.06	0.35	0.37	0.43	0.41	0.37	0.39	0.04	0.43	0.44	0.40	0.39	0.33	0.38	0.41	0.03	0.44
6	0.03	0.37	0.43	0.05	0.38	0.02	0.00	0.41	0.38	0.01	0.02	0.43	0.00	0.39	0.40	0.00	0.03	0.40
...																		
10^6	0.04	0.34	0.09	0.40	0.01	0.38	0.05	0.03	0.03	0.39	0.45	0.41	0.04	0.43	0.07	0.10	0.40	0.39

→

Debarcoded samples

	Target ID isotopes						Sample ID isotopes															
	^{159}Tb	^{160}Gd	^{161}Dy	^{162}Dy	^{163}Dy	^{164}Dy	^{165}Ho	^{166}Er	^{167}Er	^{168}Er	^{169}Tm	^{170}Er	^{171}Yb	^{172}Yb	^{173}Yb	^{174}Yb	^{175}Lu	^{176}Yb	Target ID sum	SampleID sum	Target barcode	Sample barcode
1	1	1	1	0	0	0	0	0	0	0	0	0	1	1	1	1	1	1	3	6	1	924
2	0	0	0	1	1	1	1	1	1	1	1	1	0	0	0	0	0	0	3	6	20	1
3	0	1	1	1	1	1	1	1	0	0	1	0	1	0	1	1	0	0	5	6	n/a	160
4	1	1	0	0	0	1	1	1	1	0	1	0	0	1	0	1	0	1	3	7	4	n/a
5	0	0	1	1	1	1	1	1	0	1	1	1	1	1	1	1	0	1	4	10	n/a	n/a
6	0	1	1	0	1	0	0	1	1	0	0	1	0	1	1	0	0	1	3	6	12	566
...																						
10^6	0	1	0	1	0	1	0	0	0	1	1	1	0	1	0	0	1	1	3	3	15	856

Step 10. Downstream analysis. A table of the levels of IgG (isotope ^{197}Au) and IgM (isotope ^{115}In) per Target and Sample ID is generated and then analyzed.

	Sample ID	Target ID	IgG ^{197}Au	IgM ^{115}In
**1**	924	12	0.22	0.06
**2**	1	6	0.30	0.14
**3**	160	12	0.23	0.04
**6**	566	11	0.44	0.34
...				
**10^6**	856	1	0.25	0.16

<u>Supplementary Note 3:</u> The numbers to control and optimize the stoichiometry of biotin to streptavidin at the population level

The polystyrene used in this work (Spherotech, # SVP-30-5) have a size range of 3 to 3.4 *μ*m diameter and they are at 0.5% w/v. Therefore, 1 mL (5 mg) of beads have a binding capacity of 29.41 to 66.67 *μ*g of biotinylated BSA:

						Typical binding capacity	
Bead Diameter (μm)	Polymer Density	Polymer Weight (g)	Surface area (cm^2)	Particle Number ($x10^9$)	Surface to surface of 1 mg 4 μm beads	Low range	High range
3	1.05	0.001	19.05	0.067	1.33	6.67	13.33
3.4	1.05	0.001	16.81	0.046	1.18	5.88	11.76
4	1.05	0.001	14.29	0.028	1.00	5.00*	10.00*
3	1.05	0.005	95.24	0.337	6.67	33.33	66.67
3.4	1.05	0.005	84.03	0.232	5.88	29.41	58.82
4	1.05	0.005	71.43	0.142	5.00	25.00	50.00
3	1.05	0.01	190.48	0.674	13.33	66.67	133.33
3.4	1.05	0.01	168.07	0.463	11.76	58.82	117.65
4	1.05	0.01	142.86	0.284	10.00	50.00	100.00

*One mg of a 4 *μ*m Spherotech streptavidin particles typically binds 5 to 10 *μ*g of biotinylated antibody. We assume the binding capacity for biotinylated BSA is similar to the binding capacity of biotinylated antibody given the variability of the biotinylation process.

Particle number = $(6W/3.14PD^3) \times 10^{12}$
Total surface area (cm^2) of bead population = $(6W/PD) \times 10^4$

Where,
W = Polymer Weight (in grams)
P = Polymer Density (polystyrene density is 1.05)
D = Particle Diameter (in *μ*m)

The description of polystyrene particles can be found in the Spherotech website.

Captions for the supplementary tables

Supplementary Table 1 l The 19 distinct SARS-CoV-2 protein variants and subunits, related to Fig. 4.

Supplementary Table 2 l Samples from the FIND study and associated metadata, related to Fig. 4.

Supplementary Table 3 l Table key for target ID identification, related to the automatic bead debarcoding.

Supplementary Table 4 l Table key for sample ID identification, related to the automatic bead debarcoding.
