# Supplementary material for: High-throughput multiplexed serology via the mass-spectrometric analysis of isotopically barcoded beads
Source: Nat Biomed Eng. 2025 Feb 12;9(7):1117–28. doi: 10.1038/s41551-025-01349-0 (PMC12270904; doi:10.1038/s41551-025-01349-0)
Supplement: Supplementary file 2 — Reporting Summary [file 41551_2025_1349_MOESM2_ESM.pdf]

nature portfolio

Corresponding author(s): Xavier Rovira Clave

Last updated by author(s): Aug 23, 2024

Reporting Summary

Nature Portfolio wishes to improve the reproducibility of the work that we publish. This form provides structure for consistency and transparency in reporting. For further information on Nature Portfolio policies, see our Editorial Policies and the Editorial Policy Checklist.

Statistics

For all statistical analyses, confirm that the following items are present in the figure legend, table legend, main text, or Methods section.

n/a	Confirmed	
☐	☒	The exact sample size (n) for each experimental group/condition, given as a discrete number and unit of measurement
☐	☒	A statement on whether measurements were taken from distinct samples or whether the same sample was measured repeatedly
☒	☐	The statistical test(s) used AND whether they are one- or two-sided<br>*Only common tests should be described solely by name; describe more complex techniques in the Methods section.*
☒	☐	A description of all covariates tested
☒	☐	A description of any assumptions or corrections, such as tests of normality and adjustment for multiple comparisons
☐	☒	A full description of the statistical parameters including central tendency (e.g. means) or other basic estimates (e.g. regression coefficient) AND variation (e.g. standard deviation) or associated estimates of uncertainty (e.g. confidence intervals)
☒	☐	For null hypothesis testing, the test statistic (e.g. F, t, r) with confidence intervals, effect sizes, degrees of freedom and P value noted<br>*Give P values as exact values whenever suitable.*
☒	☐	For Bayesian analysis, information on the choice of priors and Markov chain Monte Carlo settings
☒	☐	For hierarchical and complex designs, identification of the appropriate level for tests and full reporting of outcomes
☒	☐	Estimates of effect sizes (e.g. Cohen's d, Pearson's r), indicating how they were calculated

Our web collection on statistics for biologists contains articles on many of the points above.

Software and code

Policy information about availability of computer code

Data collection	For mass-cytometry experiments, the software used to collect the data was DVS Sciences Cytof instrument control software from Fluidigm.
Data analysis	Beads were debarcoded using a custom R script. Data were analysed with R (3.6.2), and plots were created using the ggplot2 R package (3.4.3). All figures were prepared using Adobe Illustrator (24.2.3).

For manuscripts utilizing custom algorithms or software that are central to the research but not yet described in published literature, software must be made available to editors and reviewers. We strongly encourage code deposition in a community repository (e.g. GitHub). See the Nature Portfolio guidelines for submitting code & software for further information.

Data

Policy information about availability of data

All manuscripts must include a data availability statement. This statement should provide the following information, where applicable:

- Accession codes, unique identifiers, or web links for publicly available datasets
- A description of any restrictions on data availability
- For clinical datasets or third party data, please ensure that the statement adheres to our policy

The data supporting the findings of the study are provided within the paper and its supplementary information. Raw data related to this manuscript can be accessed at https://zenodo.org/records/10822264.

Research involving human participants, their data, or biological material

Policy information about studies with human participants or human data. See also policy information about sex, gender (identity/presentation), and sexual orientation and race, ethnicity and racism.

Reporting on sex and gender	Sex and gender information for the FIND COVID study was determined on the basis of self-reporting, and reported in Supplementary table 2 as de-identified data at the individual level. Sex and gender information was not collected for the other samples. No studies based on sex and gender were performed in this work.
Reporting on race, ethnicity, or other socially relevant groupings	Ethnicity information for the FIND COVID study was determined on the basis of self-reporting, and reported in Supplementary table 2 as de-identified data at the individual level. Ethnicity information was not collected for the other samples. No studies based on ethnicity were performed in this work.
Population characteristics	Age, weight, height, schooling level, household information, COVID-19 diagnosis by PCR, and vaccination status for the FIND COVID study were determined on the basis of self-reporting, and are provided in Supplementary table 2 as de-identified data at the individual level.
Recruitment	Some samples were collected from COVID-19 convalescent plasma donors who donated plasma at the Stanford Blood Center between April 2020 and May 2020. Other samples were from the FIND COVID study, a CDC-funded longitudinal cohort of individuals recently diagnosed with SARS-CoV-2 infections in the San Francisco Bay Area, initiated in August 2020.
Ethics oversight	The Stanford samples study was approved by the Stanford University Institutional Review Board (Protocols IRB-13952, IRB-48973, and IRB-55689). The FIND COVID study was approved by the UCSF Institutional Review Board (Protocol IRB# 20–30388) and given a designation of public health surveillance according to federal regulations as summarized in 45 CFR 46.102(d)(1)(2).

Note that full information on the approval of the study protocol must also be provided in the manuscript.

Field-specific reporting

Please select the one below that is the best fit for your research. If you are not sure, read the appropriate sections before making your selection.

☒ Life sciences ☐ Behavioural & social sciences ☐ Ecological, evolutionary & environmental sciences

For a reference copy of the document with all sections, see nature.com/documents/nr-reporting-summary-flat.pdf

Life sciences study design

All studies must disclose on these points even when the disclosure is negative.

Sample size	Most experiments were aimed at describing a new technology; thus, no sample-size calculation was performed. Sample sizes were selected to properly demonstrate and validate technical performance. For the experiments related to the FIND COVID study, the sample size of 542 is higher than those in comparable work.
Data exclusions	No data were excluded.
Replication	All replication attempts were successful.
Randomization	Samples were randomly distributed into 96-well plates.
Blinding	Blinding was not necessary because the samples were obtained de-identified.

Reporting for specific materials, systems and methods

We require information from authors about some types of materials, experimental systems and methods used in many studies. Here, indicate whether each material, system or method listed is relevant to your study. If you are not sure if a list item applies to your research, read the appropriate section before selecting a response.

Materials & experimental systems

n/a	Involved in the study	
☐	☒	Antibodies
☒	☐	Eukaryotic cell lines
☒	☐	Palaeontology and archaeology
☒	☐	Animals and other organisms
☒	☐	Clinical data
☒	☐	Dual use research of concern
☒	☐	Plants

Methods

n/a	Involved in the study	
☒	☐	ChIP-seq
☒	☐	Flow cytometry
☒	☐	MRI-based neuroimaging

Antibodies

Antibodies used	Information of the antibodies used in this study is also detailed in Methods:<br>human anti-Spike S1 (Invivogen, #srbd-mab1)<br>MACS anti-IgG microbeads (Miltenyi Biotec, #130-047-501)<br>anti-IgG-alexa647 (Thermo Fischer Scientific, #A21445)<br>gold anti-IgG antibody (Nanoprobes, #2053)<br>anti-IgM antibody (BioLegend, #314502)<br>horseradish peroxidase-conjugated goat anti-human IgG (Thermo Fisher, #62-8420)
Validation	Antibody–antigen interaction experimentally confirmed in vitro.
